# Ischemia-Reperfusion Injury After Posterior Cervical Laminectomy

**DOI:** 10.7759/cureus.18298

**Published:** 2021-09-26

**Authors:** Matea Malinovic, James Walker, Felecia Lee

**Affiliations:** 1 Anesthesiology, University of Kansas School of Medicine, Wichita, USA

**Keywords:** paralysis, spinal decompression, myelomalacia, white cord, ischemia-reperfusion injury

## Abstract

Ischemia-reperfusion injury is a rare but serious complication encountered after spinal decompression surgery. This is only the 11th case reported in the literature. There is no current mainstay of treatment; however, several therapies have been studied. This case presents a patient with myelomalacia who underwent posterior laminectomy and developed diffuse cord edema with postoperative quadriplegia. Ischemia-reperfusion injury is believed to be mediated by oxidative and nitrosative stress leading to protein degradation and lipid peroxidation. It is characterized by myelomalacia in a chronically ischemic spinal cord and hyperintensity on T2-weighted MRI after decompression. Treatment has involved steroids and rehabilitation, and outcomes have ranged from minor improvement to full recovery. Novel treatment options have shown promise in animal models.

## Introduction

Posterior decompression via laminectomy is a commonly performed procedure for patients who present with cervical compressive myelopathy. Complications related to the procedure include cervical palsies, infection, and epidural hematoma [1]. One rare and serious complication that can arise from surgery is ischemia-reperfusion injury. Described as “white cord syndrome” due to its appearance on T2-weighted MRI, this condition has been described previously in only 10 case reports [2-10]. While defined as a diagnosis of exclusion, this condition is hypothesized to be mediated by reactive oxygen species, proinflammatory mediators such as cytokines and chemokines, and signaling cascades involving nitric oxide and complement following the hyperemia caused by the restoration of blood flow to a chronically ischemic spinal cord [11-13]. This condition can cause transient or permanent paralysis in patients. This case report summarizes an instance of permanent paralysis due to suspected ischemia-reperfusion injury after posterior decompression in a patient with achondroplasia and chronic cervical compression. Written informed consent was obtained on behalf of the patient. This article has been presented as a virtual poster at the University of Kansas School of Medicine - Wichita 28th Annual Research Forum on April 17, 2020, and the Midwest Anesthesia Residents’ Conference on April 17, 2021.

## Case presentation

**Figure 1:**
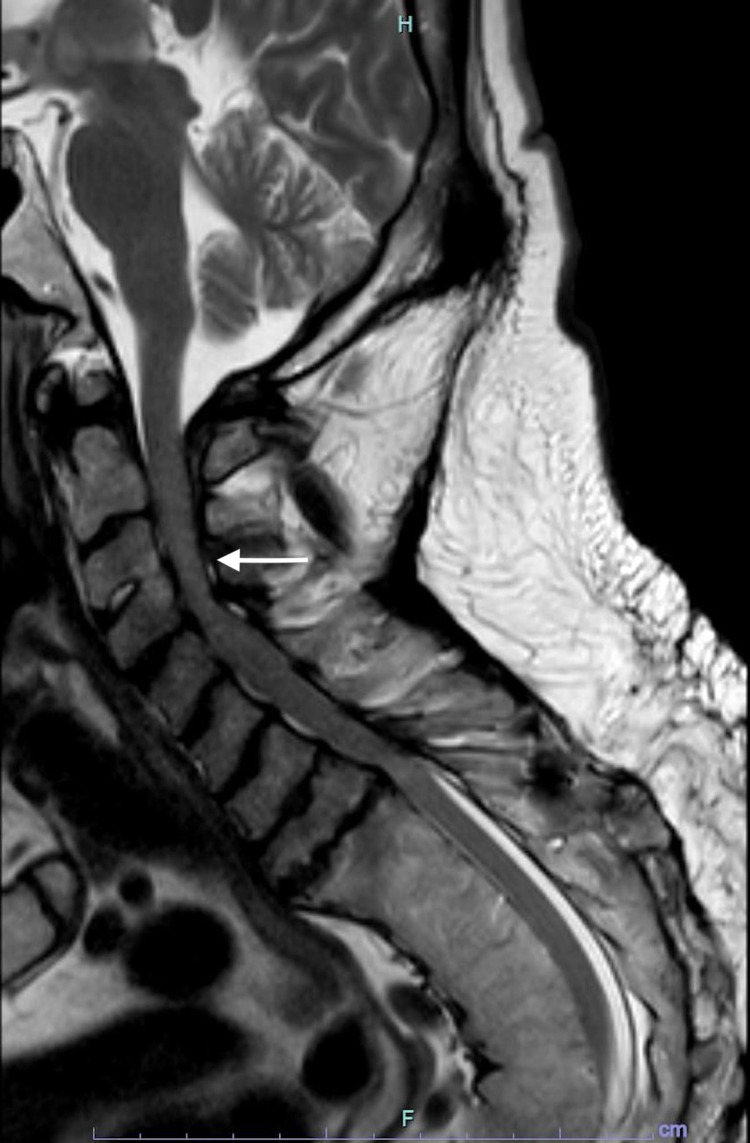
Preoperative MRI. Preoperative MRI indicating mild cord atrophy from C2 to C4, moderate stenosis at C2-C3 and C5-C7, and severe stenosis at C3-C5 (indicated by the arrow). In addition, disc herniation was noted at C4-C5 and C6-C7.

A 46-year-old male with a six-month history of fatigue, frequent sharp shooting neck pain, numbness with weakness in the bilateral upper extremities, and gait abnormalities presented to the neurosurgical clinic for evaluation of his severe cervical stenosis. The patient had a past medical history of achondroplasia, cervical stenosis, cervical disc degeneration, club foot, obstructive sleep apnea, and scoliosis. His surgical history included numerous surgical procedures including a prior thoracic scoliosis fusion. Preoperative imaging noted mild cord atrophy from C2 to C4, moderate stenosis at C2-C3 and C5-C7, and severe stenosis at C3-C5 (Figure 1). In addition, disc herniation was noted at C4-C5 and C6-C7.

At initial evaluation on the day of surgery, he had 5/5 motor strength in bilateral upper extremities, weakness in bilateral lower extremities necessitating crutches, and grossly intact sensation in all four extremities. He endorsed numbness and had a negative Hoffmann’s sign in his bilateral upper extremities. A left radial arterial line was placed and mean arterial pressure (MAP) was maintained greater than 80 mmHg throughout the case with a norepinephrine drip. Intubation was performed with cervical spine precautions, with his neck maintained in the neutral position during intubation using a GlideScope (Verathon Inc., Bothell, WA) and while turned to the prone position with the head in a horseshoe throughout the case. Neuromonitoring was not utilized due to the surgeon's preference. The patient underwent C2-T2 posterior decompression via laminectomy. Hemostasis was achieved prior to closure. There were no surgical or anesthetic complications noted intraoperatively.

**Figure 2:**
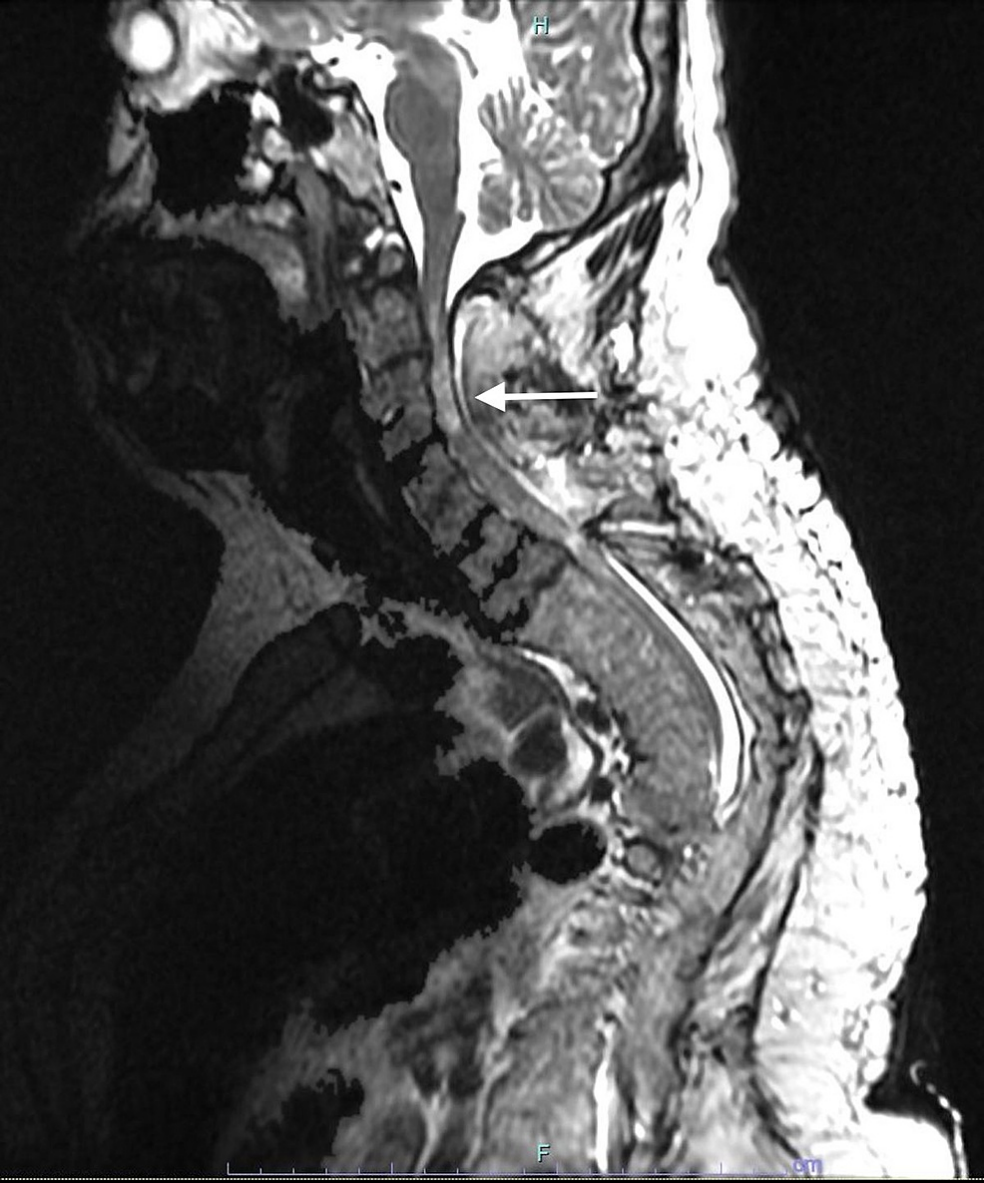
Postoperative MRI. Postoperative MRI of the cervical spine revealed decompressive laminectomy with substantial C2-C5 cord edema (indicated by the arrow) without pathological diffusion restriction to suggest irreversible ischemia.

Upon evaluation in the post-anesthesia care unit, the patient had a motor function in bilateral thumbs with preserved sensation down to the umbilicus. A 250 mL 5% albumin bolus and 100 mcg of phenylephrine were administered prior to restarting a norepinephrine drip to maintain MAP greater than 80 mmHg. Hoffmann and Babinski's signs were both negative bilaterally and there was no clonus. MRI of the cervical spine performed slightly over four hours after arrival to the post-anesthesia care unit revealed decompressive laminectomy with substantial C2-C5 central cord edema without pathological diffusion restriction to suggest irreversible ischemia (Figure 2). While no hematoma or mass effects were noted on the cord, there was progressive signal abnormality at T1-T2. Though lack of diffusion abnormality on MRI does not rule out ischemia, maintenance of MAP greater than 80 mmHg throughout the case makes ischemia a much less likely cause of the postoperative neurologic changes.

Measures were initiated to reduce the edema seen on MRI, including dexamethasone and hypertonic saline boluses. While research on the benefit of steroid use for acute spinal cord injury is lacking, dexamethasone and hypertonic saline were started to mitigate the effect of spinal cord edema. None of these measures resulted in significant improvement. He continued to have only minimal bilateral thumb movement and his sensory deficit regressed to the nipple line on postoperative day (POD) one. On POD three, he regained flexion in his left upper extremity and bicep movement and some sensation of pain below the level of the nipples. Steroids and hypertonic saline were discontinued by the surgeon on POD three as both had been attempted based on theoretical benefit but neither had led to any clinical improvement. After a prolonged hospital stay, he failed to regain any further motor or sensory function.

## Discussion

Ten cases of ischemia-reperfusion injury have been described in the literature to date [2-10]. Of these, three cases involved anterior [2-4] cervical decompression and fusion, while seven cases involved a posterior [4-10] approach (Table 1). All patients in these case reports had varying degrees of paresis or plegia (hemiplegia to complete quadriplegia) postoperatively. Their deficits presented either immediately postoperatively or up to 24 hours later, except for the two patients described by Singh et al. [5] (Table 1). The authors postulated that the delayed onset could have been due to the patients having a pre-existing diagnosis of hypertension leading to endothelial damage and a decrease in nitric oxide due to aging, which may have led to subacute reperfusion and therefore delayed onset [5]. All patients were treated with steroids and extensive inpatient rehabilitation. Recovery ranged from minor improvement of neurological symptoms to complete recovery over a period of a few months (Table 1).

**Table 1:** Prior clinical cases involving ischemia-reperfusion and white cord syndrome. ACDF - anterior cervical discectomy and fusion; PCDF - posterior cervical discectomy and fusion; SSEP - somatosensory evoked potential; MEP - motor evoked potential; POD - postoperative day; NASCIS - National Acute Spinal Cord Injury Studies.

Year	Demographics	Surgery	Neuromonitoring	Positioning	Myelomalacia preoperatively, T2 hyperintensity postoperatively	Timing of deficit	Treatment	Outcome	Reference
2013	59M	C4-C5, C5-C6 ACDF	SSEP, MEP	Not addressed	Yes	Intraoperative	Further decompression with corpectomy, steroids	Nurick 4	Chin et al. [2]
2017	64M	C3-C4, C5-C6 ACDF	SSEP, MEP	Not addressed	Yes	Intraoperative	NASCIS III - 2 days of steroids	Nurick 4	Giammalva et al. [3]​​​​​​
2018	68M	C4-C7 PCDF	SSEP, MEP	Not addressed	Yes	Intraoperative	Further decompression, steroids	Nurick 4	Antwi et al. [6]​​​​​​
2018	79M	C2-C7 PCDF	States use of neuromonitoring	Not addressed	Yes	Postoperatively - 24 hours	Further decompression, NASCIS III for 2 days	Nurick 4	Papaioannou et al. [8]​​​​​​​​​​
2018	51F	C2-C5 PCDF	Not addressed	Not addressed	Yes	Immediately postoperative	Steroids	Weaned off the ventilator, no other improvement	Vinodh et al. [10]​​​​​​​​​​​​​
2019	41M	C1-C2 PCDF	SSEP, MEP	Not addressed	Yes	Intraoperative	Steroids	Full recovery	Wiginton et al. [7]​​​​​​​​​​​​​
2020	79M	C2-C6 PCDF	SSEP, MEP	Not addressed	Yes	Intraoperative	Steroids	Nurick 1	Mathkour et al. [9]​​​​​​​​​​​​​
2020	49F	C6-C7 ACDF	Not addressed	Not addressed	Yes	Immediately postoperative	Laminoplasty at C4-C7 levels, steroids	Full recovery	Jun et al. [4]​​​​​​​​​​​​​
2021	59F	C3-C6 PCDF	Not addressed	Not addressed	Yes	POD 2	Steroids	Stand independently, grade 4 muscle strength in all limbs	Singh et al. [5]​​​​​​​​​​​
2021	66F	C6-C7 ACDF with anterior plate fixation	Not addressed	Supine, Mayfield skull clamp, cervical spine in slight extension	Yes	POD 8	Steroids	Improvement, walk with assisted	Singh et al. [5]​​​​​​​​​​​​​​​​​​

Our case shares some common characteristics with those in the existing literature, described in Table 1. None of the 11 cases describe any mechanical compromise or compression to the spinal cord during the operation itself [2-10]. The common themes of myelomalacia evident on preoperative MRI and hyperintensity noted on postoperative MRI are seen in all cases. This is unique from severe cervical myelopathy patients in that the spinal cord is edematous postoperatively. Our patient exhibited severe neurological impairment as noted in the case described by Vinodh et al. [10]. However, the patient described in that case did improve enough to be able to be weaned from the ventilator.

Ischemia-reperfusion is hypothesized to be mediated by oxidative and nitrosative stress [11]. Through these reactions, effects such as protein degradation, lipid peroxidation, and inflammation occur, leading to cell death. Strategies for attenuating these effects have been studied in phase III clinical trials and include methylprednisolone, naloxone, tirilazad, and the monosialoganglioside GM1, with each showing modest benefit to patients with spinal cord injuries [14]. In addition, dexamethasone has been identified as an attenuation strategy against lipid peroxidation seen in spinal cord injury [14]. Conflicting evidence exists on steroid therapy, with currently no class I or II recommendations [15]. In fact, steroids have been associated with serious side effects such as hyperglycemia and increased rates of infection [15]. Dexamethasone was used in an attempt to mitigate spinal cord edema seen on postoperative MRI. Neurologic damage in this situation is not associated with an initial traumatic spinal cord injury but is instead related to reperfusion with proinflammatory mediators. Steroids depress the inflammatory response that causes the damage.

A study by Vidal et al. in a mouse model has demonstrated that delaying decompressive surgery for compressive myelopathy led to an increased risk and more serious manifestation of reperfusion injury [16]. Prolonged ischemia of the spinal cord due to delay in decompressive surgery leads to increased blood flow after decompression [17]. While Vidal et al. did not conclude a time frame for symptoms in humans to be officially classified as early or late prior to decompression, the unadjusted model used in the study defined the time frame as six months [16]. The patient in this case report presented with over a six-month history of symptoms. Earlier decompressive surgery may have reduced his risk of developing ischemia-reperfusion injury and the severity of his neurological deficits. Delayed decompression may make reperfusion injury more likely. However, since surgery will likely remain the last option, anticipating this risk may allow providers to initiate therapies to preemptively modify this risk.

Some of the other strategies that have shown potential in mitigating effects of ischemia-reperfusion injury in animal models include riluzole (sodium glutamate antagonist), thymoquinone, and hydroxysafflor yellow A [17-19]. They have all led to decreased inflammation and increased antioxidant activity, with improved postoperative functional status. In addition, propofol has been shown to attenuate spinal cord injury by decreasing inflammation through the inhibition of nuclear factor kappa B (NF-kB) pathways, decreasing the expression of proinflammatory mediators, and improving maintenance of the blood-spinal cord barrier [20]. This could alter the anesthetic plan for decompressive spinal surgeries to exclusively involve intravenous anesthetic with propofol instead of volatile gases.

## Conclusions

Ischemia-reperfusion injury of the spinal cord is a rare and serious complication seen after spinal decompressive surgeries. It is a diagnosis of exclusion. Oxidative damage leading to cell apoptosis and necrosis has been implicated in the pathophysiology of this syndrome. This case highlights the 11th case seen in the literature surrounding this condition, though the true incidence of this phenomenon may be much higher. Like prior case reports, our patient was treated with steroids and rehabilitation. While there is no mainstay of treatment after ischemia-reperfusion injury, several new modalities have shown promising results in animal model research. Further animal studies need to be performed using these strategies to evaluate their potential synergistic relationships prior to use in human subjects.

## References

[1] Wang T, Tian XM, Liu SK, Wang H, Zhang YZ, Ding WY (2017). Prevalence of complications after surgery in treatment for cervical compressive myelopathy: a meta-analysis for last decade. Medicine (Baltimore).

[2] Chin KR, Seale J, Cumming V (2013). "White cord syndrome" of acute tetraplegia after anterior cervical decompression and fusion for chronic spinal cord compression: a case report. Case Rep Orthop.

[3] Giammalva GR, Maugeri R, Graziano F, Gulì C, Giugno A, Basile A, Iacopino DG (2017). White cord syndrome after non-contiguous double-level anterior cervical decompression and fusion (ACDF): a “no reflow phenomenon”?. Interdiscip Neurosurg.

[4] Jun DS, Baik JM, Lee SK (2020). A case report: white cord syndrome following anterior cervical discectomy and fusion: importance of prompt diagnosis and treatment. BMC Musculoskelet Disord.

[5] Singh RD, Arts MP, de Ruiter GC (2021). Delayed-onset white cord syndrome after anterior and posterior cervical decompression surgery for symptomatic ossification of spinal ligaments: illustrative cases. [PREPRINT]. J Neurosurg.

[6] Antwi P, Grant R, Kuzmik G, Abbed K (2018). "White cord syndrome" of acute hemiparesis after posterior cervical decompression and fusion for chronic cervical stenosis. World Neurosurg.

[7] Wiginton JG 4th, Brazdzionis J, Mohrdar C, Sweiss R, Lawandy S (2019). Spinal cord reperfusion injury: case report, review of the literature, and future treatment strategies. Cureus.

[8] Papaioannou I, Repantis T, Baikousis A, Korovessis P (2019). Late-onset "white cord syndrome" in an elderly patient after posterior cervical decompression and fusion: a case report. Spinal Cord Ser Cases.

[9] Mathkour M, Werner C, Riffle J, Scullen T, Dallapiazza RF, Dumont A, Maulucci C (2020). Reperfusion "white cord'' syndrome in cervical spondylotic myelopathy: does mean arterial pressure goal make a difference? Additional case and literature review. World Neurosurg.

[10] Vinodh VP, Rajapathy SK, Sellamuthu P, Kandasamy R (2018). White cord syndrome: a devastating complication of spinal decompression surgery. Surg Neurol Int.

[11] Fatima G, Sharma VP, Das SK, Mahdi AA (2015). Oxidative stress and antioxidative parameters in patients with spinal cord injury: implications in the pathogenesis of disease. Spinal Cord.

[12] Smith PD, Puskas F, Meng X (2012). The evolution of chemokine release supports a bimodal mechanism of spinal cord ischemia and reperfusion injury. Circulation.

[13] Cowled P, Fitridge R (2011). Pathophysiology of reperfusion injury. Mechanisms of Vascular Disease: A Reference Book for Vascular Specialists.

[14] Hall ED, Springer JE (2004). Neuroprotection and acute spinal cord injury: a reappraisal. NeuroRx.

[15] Hurlbert RJ, Hadley MN, Walters BC (2015). Pharmacological therapy for acute spinal cord injury. Neurosurgery.

[16] Vidal PM, Karadimas SK, Ulndreaj A (2017). Delayed decompression exacerbates ischemia-reperfusion injury in cervical compressive myelopathy. JCI Insight.

[17] Karadimas SK, Laliberte AM, Tetreault L, Chung YS, Arnold P, Foltz WD, Fehlings MG (2015). Riluzole blocks perioperative ischemia-reperfusion injury and enhances postdecompression outcomes in cervical spondylotic myelopathy. Sci Transl Med.

[18] Gökce EC, Kahveci R, Gökce A (2016). Neuroprotective effects of thymoquinone against spinal cord ischemia-reperfusion injury by attenuation of inflammation, oxidative stress, and apoptosis. J Neurosurg Spine.

[19] Shan LQ, Ma S, Qiu XC (2010). Hydroxysafflor yellow A protects spinal cords from ischemia/reperfusion injury in rabbits. BMC Neurosci.

[20] Xie LJ, Huang JX, Yang J, Yuan F, Zhang SS, Yu QJ, Hu J (2017). Propofol protects against blood-spinal cord barrier disruption induced by ischemia/reperfusion injury. Neural Regen Res.

